# PKC Inhibition Improves Human Penile Vascular Function and the NO/cGMP Pathway in Diabetic Erectile Dysfunction: The Role of NADPH Oxidase

**DOI:** 10.3390/ijms25063111

**Published:** 2024-03-07

**Authors:** Mariam El Assar, José M. La Fuente, Patricia Sosa, Argentina Fernández, Augusto J. Pepe-Cardoso, Juan I. Martínez-Salamanca, Leocadio Rodríguez-Mañas, Javier Angulo

**Affiliations:** 1 Fundación para la Investigación Biomédica del Hospital de Getafe, 28905 Getafe, Spain; patricia.sosa@iisgetafe.com; 2 Centro de Investigación Biomédica en Red de Fragilidad y Envejecimiento Saludable (CIBERFES), Instituto de Salud Carlos III, 28029 Madrid, Spain; leocadio.rodriguez@salud.madrid.org; 3 Instituto de Investigación IdiPaz, 28029 Madrid, Spain; 4 Serviço de Urologia, Centro Hospitalar e Universitário de Santo António (CHUdSA), 4099-001 Porto, Portugal; lafuentecarvalho@gmail.com; 5 Servicio de Histología-Investigación, Unidad de Investigación Traslacional en Cardiología—IRYCIS/UFV, Hospital Universitario Ramón y Cajal, 28034 Madrid, Spain; argentina.fernandez@hrc.es; 6 Serviço de Urologia, Hospital Fernando da Fonseca, 2720-276 Amadora, Portugal; apepecardoso@gmail.com; 7 Servicio de Urología, Hospital Universitario Puerta de Hierro, 28222 Madrid, Spain; jims09@me.com; 8 Servicio de Geriatría, Hospital Universitario de Getafe, 28905 Getafe, Spain

**Keywords:** diabetes, erectile dysfunction, human corpus cavernosum, human penile arteries, endothelial dysfunction, NO/cGMP pathway, protein kinase C, NADPH oxidase

## Abstract

Erectile dysfunction (ED) is a frequent and difficult-to-treat condition in diabetic men. Protein kinase C (PKC) is involved in diabetes-related vascular and cavernosal alterations. We aimed to evaluate the role of PKC in endothelial dysfunction and NO/cGMP impairment associated with diabetic ED in the human corpus cavernosum (CC) and penile resistance arteries (PRAs) and the potential mechanisms involved. Functional responses were determined in the CC and PRAs in patients with non-diabetic ED and diabetic ED undergoing penile prosthesis insertion. PKC activator 12,13-phorbol-dibutyrate (PDBu) impaired endothelial relaxations and cGMP generation in response to acetylcholine in the CC from non-diabetic ED. PDBu also impaired responses to a PDE5 inhibitor, sildenafil, in non-diabetic ED patients. Conversely, a PKC inhibitor, GF109203X, improved endothelial, neurogenic, and PDE5-inhibitor-induced relaxations and cGMP generation only in the CC in diabetic ED patients. Endothelial and PDE5-inhibitor-induced vasodilations of PRAs were potentiated only in diabetes. Improvements in endothelial function in diabetes were also achieved with a specific inhibitor of the PKCβ2 isoform or an NADPH-oxidase inhibitor, apocynin, which prevented PDBu-induced impairment in non-diabetic patients. PKC inhibition counteracted NO/cGMP impairment and endothelial dysfunction in diabetes-related ED, potentially improving response to PDE5 inhibition.

## 1. Introduction

Diabetes mellitus (DM) has become a significant public health concern, with an estimated 8.8% of the adult population being affected in 2015. Projections for the future are even more worrisome, indicating that by 2040, the global proportion of adults with DM is anticipated to rise to 10.4% [1]. Diabetes stands out as a primary risk factor for the onset of erectile dysfunction (ED). In fact, the prevalence of ED in patients with DM is approximately 3.5-fold higher than that in non-diabetic patients [2]. In addition, diabetic men with ED are less responsive to the current first-line therapy with phosphodiesterase type-5 (PDE5) inhibitors [3], which seriously affects their quality of life [4]. Therefore, there is an emerging need to enhance our comprehension of the underlying molecular mechanisms and to develop alternative targets for the treatment of ED related to diabetes.

Alterations in neurovascular function appear to play a pivotal role in the pathophysiology of erectile dysfunction related to diabetes. In line with this, evidence showed that diabetic men suffering from ED manifested decreased endothelium-dependent and neurogenic relaxations in the corpus cavernosum and penile resistance arteries in contrast to their non-diabetic counterparts [5]. Furthermore, defective endothelial, neurogenic, and PDE5-inhibitor-induced relaxations were also observed in the corpus cavernosum of streptozotocin-induced diabetic rats when compared to non-diabetic animals [6]. Vasorelaxant responses mediated by endothelium-derived hyperpolarization were also reduced in penile arteries from Zucker diabetic fatty rats [7] and in diabetic men with ED [8].

These alterations in vascular function could be attributed in part to the disruption of the nitric oxide (NO)/cyclic guanosine monophosphate (cGMP) pathway which is a pivotal mediator in penile smooth muscle relaxation and erection. Indeed, NO contributes to endothelium-dependent and neurogenic relaxation in penile vascular tissue. In line with this, impaired endothelium-dependent relaxation mediated by NO has been observed in penile vascular tissue from diabetic subjects [8]. Decreased activity of NO has been reported in corpus cavernosum tissues derived from diabetic patients [9]. Furthermore, the presence of diabetes condition exacerbated the functional deficiency of NO/cGMP related to ED in human corpus cavernosum (CC) and penile resistance arteries (PRA) and reduced cGMP content in penile tissues when compared to non-diabetic patients [5].

Vascular dysfunction related to diabetes and hyperglycemia is due to multiple molecular mechanisms, including the hyperactivation of protein kinase C (PKC) and oxidative stress [10,11]. PKC is a family of serine/threonine protein kinases encompassing a family of isoforms, including classical PKC (α, β, and γ), as well as other novel PKC and atypical PKC isoforms [11]. Functional studies have suggested a potential role of PKC in diabetes-related cavernosal alterations. In this sense, an enhancement of endothelium-dependent and neurogenic relaxations of the corpus cavernosum in diabetic mice was observed following chronic inhibition of PKC beta [12]. Furthermore, functional studies have reported the contribution of PKC overactivity to the defective endothelium-dependent vasodilation in tissue from diabetic subjects [13]. In fact, PKC has been proposed as a potential therapeutic target for endothelial dysfunction in diabetes [14].

Oxidative stress plays a crucial role in the development of ED in diabetic subjects [15]. The reciprocal interaction between oxidative stress and PKC activation is of significance, with reactive oxygen species (ROSs) being thought to promote PKC activation; conversely, the hyperactivation of PKC has been shown to activate NADPH oxidase, resulting in increased ROS production [16,17,18] and contributing to vascular dysfunction. However, studies evaluating the role of PKC-induced NADPH activation in diabetic cavernosal tissue dysfunction are lacking. Therefore, the aim of the present study was to determine the role of PKC in penile vascular dysfunction and NO/cGMP pathway impairment related to diabetic ED and to analyze the potential role of NADPH-oxidase on PKC-related effects. Furthermore, the study aims to assess the impact of PKC inhibition on the effectiveness of PDE5 inhibitors in inducing the relaxation of penile vascular tissue in individuals with diabetic ED.

## 2. Results

The characteristics of diabetic and non-diabetic patients with ED are displayed in Table 1. No significant differences in age, cardiovascular risk factors (CVRFs), or other comorbidities were observed between the two populations of ED patients beyond the presence of diabetes. Among the diabetic ED patients, 6 had type 1 diabetes (16.7%) and 30 had type 2 diabetes (83.3%). Furthermore, organ donors without notice of erectile dysfunction (No ED) and who were free from CVRFs were also included (mean age: 41.4 ± 4.8 years old (range 18 to 68 years)).

Endothelium-dependent relaxations in response to acetylcholine (ACh) were significantly impaired in the CC in non-diabetic patients with ED with respect to that in the healthy no-ED patients. However, these responses were significantly further impaired in ED patients with diabetes with respect to the non-diabetic ED patients (Supplementary Figure S1A). This was also observed in the PRAs (Supplementary Figure S1B), confirming an exacerbated deterioration of endothelial responses in diabetic ED. On the other hand, the ACh-induced relaxations in the CC and PRAs in diabetic and non-diabetic patients with ED before and after 60 min of treatment with vehicle (0.1% DMSO) were not significantly different (Supplementary Figure S2). These allowed for the evaluation of the effects of the treatments on endothelium-dependent responses by comparing them with the previously obtained ACh-induced responses in each tissue.

### 2.1. Activation of PKC Impaired Endothelial Relaxations in the CC and PRAs in the Absence of Diabetes While PKC Inhibition Improved These Responses Only in Tissues from Diabetic Patients

**Figure 1:**
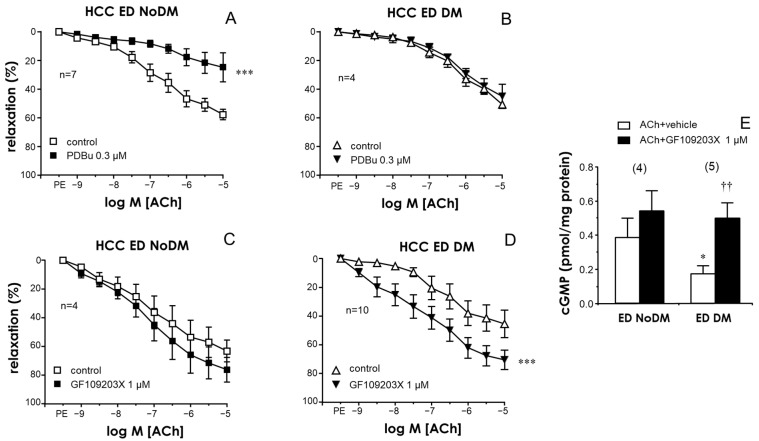
The effects of PKC inhibition on the improvement of endothelial relaxation in the human corpus cavernosum (HCC) were specifically related to the presence of diabetes. The upper panels show the effects of PKC activation with 12,13-phorbol dibutyrate (PDBu, 0.3 µM) on endothelial relaxations in response to acetylcholine (ACh, 1 nM to 10 µM) in the HCC in non-diabetic ED patients (ED NoDM) (**A**) and in diabetic patients with ED (ED DM) (**B**). The lower panels show the effects of PKC inhibition with GF109203X (1 µM) on ACh-induced responses in the HCC in ED NoDM (**C**) and ED DM (**D**). All tissues were precontracted with phenylephrine (PE, 1–10 µM). Data are expressed as the mean ± S.E.M of the percentage of relaxation. n indicates the number of patients from whom the tissues were collected. *** *p* < 0.001 according to a two-factor ANOVA. (**E**) shows the effects of GF109203X (1 µM) on the cGMP accumulation driven by ACh (10 µM) in the HCC in ED NoDM and ED DM. Data are expressed as the mean ± S.E.M. of the pmoles of cGMP normalized by the protein content of the tissue. Numbers of patients are indicated in parentheses. * *p* < 0.05 vs. ED NoDM, †† *p* < 0.01 vs. the respective data without GF109203X according to the Kruskal–Wallis test followed by Dunn’s post-hoc test.

Exposure to the activator of PKC, 12,13-phorbol dibutyrate (PDBu, 0.3 µM), caused a significant impairment of endothelium-dependent relaxations in the CC in non-diabetic ED subjects (Figure 1A). In contrast, treatment with PDBu failed to modify ACh-induced relaxation in ED patients with diabetes (Figure 1B).

Conversely, no significant effects on endothelial relaxation were observed in the CC in non-diabetic ED patients (Figure 1C) after treatment with a PKC inhibitor, GF109203X (1 µM), while a significant enhancement of ACh-induced relaxations was produced by this inhibitor of PKC in the CC in diabetic ED patients (Figure 1D). This observation could also be seen in representative tracings of ACh-induced relaxation in the CC (Supplementary Figure S3A–D). Moreover, ACh-induced cGMP accumulation was significantly reduced in the CC in ED patients with diabetes with respect to that in the non-diabetic ED subjects (Figure 1E). In addition, PKC inhibition with GF109203X (1 µM) significantly augmented ACh-induced cGMP accumulation in the CC in diabetic patients with ED, resulting in cGMP content levels after PKC inhibition that were not significantly different from those obtained in the CC in non-diabetic subjects (Figure 1E).

**Figure 2:**
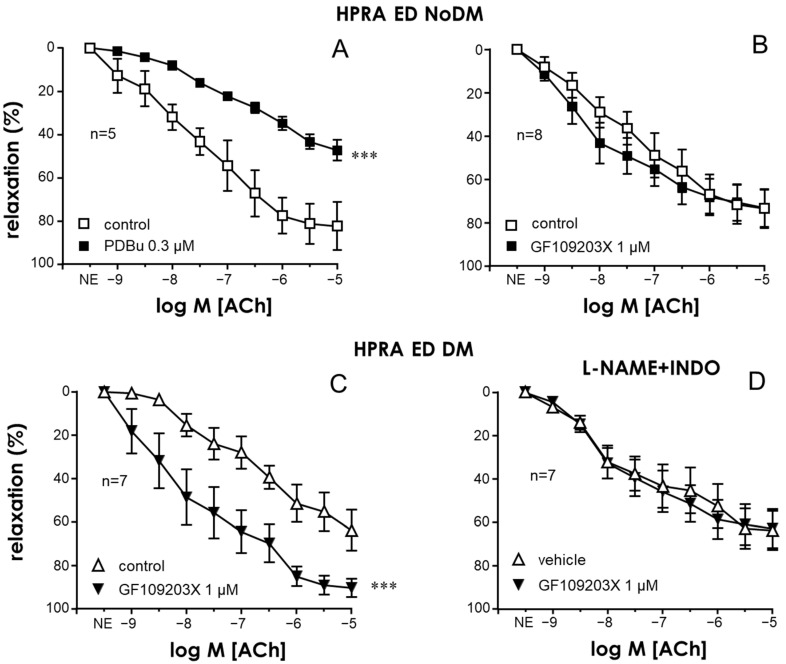
Endothelial vasodilation of human penile resistance arteries (HPRAs) in ED patients was improved through PKC inhibition only in diabetic patients. (**A**) shows the effects of PKC activation with 12,13-phorbol dibutyrate (PDBu, 0.3 µM) on endothelial vasodilations in response to acetylcholine (Ach, 1 nM to 10 µM) in the HPRAs of non-diabetic ED patients (ED NoDM). (**B**,**C**) show the effects of PKC inhibition with GF109203X (1 µM) on ACh-induced responses in the HPRAs of ED NoDM and diabetic ED patients (ED DM), respectively. (**D**) shows the influence of NO synthase and cyclooxygenase inhibition with N^G^-nitro-L-arginine methyl ester (L-NAME, 100 µM) and indomethacin (INDO, 10 µM), respectively, on the effects of GF109203X on ACh-induced responses in the HPRAs of ED DM patients. All vascular preparations were precontracted with norepinephrine (NE, 1–10 µM). Data are expressed as the mean ± S.E.M of the percentage of relaxation. n indicates the number of patients from whom the tissues were collected. *** *p* < 0.001 according to a two-factor ANOVA.

In a similar way, an activator of PKC, PDBu (0.3 µM), reduced endothelial vasodilation in the PRAs in non-diabetic patients (Figure 2A), while the PKC inhibitor GF109203X (1 µM) did not significantly modify ACh-induced vasodilations in the PRAs in non-diabetic ED patients (Figure 2B), and it caused significant potentiation of endothelial vasodilations in the PRAs in diabetic ED patients (Figure 2C). This observation can be seen in the representative tracings displayed in Supplementary Figure S3I–L. This enhancement of ACh-induced vasodilations in the PRAs of diabetic ED patients by GF109203X was not produced when the hyperpolarization component of endothelial vasodilation was isolated, i.e., when arterial segments were treated with the NO synthase inhibitor N^G^-nitro-L-arginine methyl ester (L-NAME, 100 µM) and the cyclooxygenase inhibitor indomethacin (10 µM) (Figure 2D). This points to a specific impact on the NO/cGMP pathway through PKC activation in diabetes. In fact, no significant differences in endothelial relaxation in the CC and PRAs between diabetic and non-diabetic ED patients remained after PKC inhibition with GF109203X (Supplementary Figure S4).

### 2.2. Nitrergic Neurogenic Relaxation Was Enhanced by PKC Inhibition in the CC of Diabetic ED Patients

**Figure 3:**
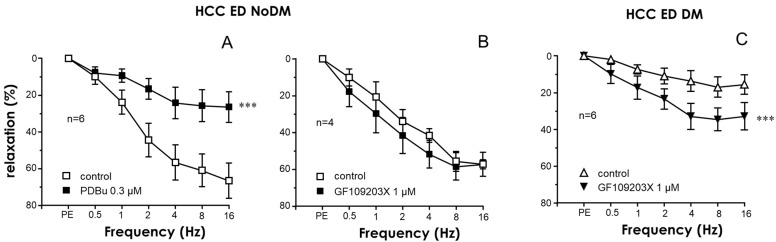
Nitrergic relaxation of the human corpus cavernosum (HCC) was enhanced by PKC inhibition specifically in diabetic patients. Effects of PKC activation with 12,13-phorbol dibutyrate (PDBu, 0.3 µM) on neurogenic nitrergic relaxations induced by electrical field stimulation (EFS, 0.5 to 16 Hz) in the HCC of non-diabetic patients with ED (ED NoDM) (**A**) and effects of PKC inhibition with GF109203X (1 µM) on nitrergic relaxations in the HCC of ED NoDM (**B**) and diabetic patients with ED (ED DM) (**C**). All tissues were precontracted with phenylephrine (PE, 1–10 µM). Data are expressed as the mean ± S.E.M of the percentage of relaxation. n indicates the number of patients from whom the tissues were collected. *** *p* < 0.001 according to a two-factor ANOVA.

Activation of PKC with PDBu (0.3 µM) caused the inhibition of nitrergic neurogenic relaxations induced by electrical field stimulation in the CC of non-diabetic ED patients (Figure 3A), while PKC inhibition with GF109203X (1 µM) failed to significantly influence electrical-field-stimulation-induced relaxations in these tissues (Figure 3B). In contrast, treatment with this PKC inhibitor significantly enhanced neurogenic nitrergic relaxations in CC strips from ED patients with diabetes (Figure 3C). Representative tracings of the effects of the PKC inhibitor on neurogenic relaxation derived from non-diabetic and diabetic subjects are shown in Supplementary Figure S3E–H.

### 2.3. PKC Inhibition Enhanced the Relaxant Efficacy of PDE5 Inhibitors in the CC and PRAs of ED Patients with Diabetes

**Figure 4:**
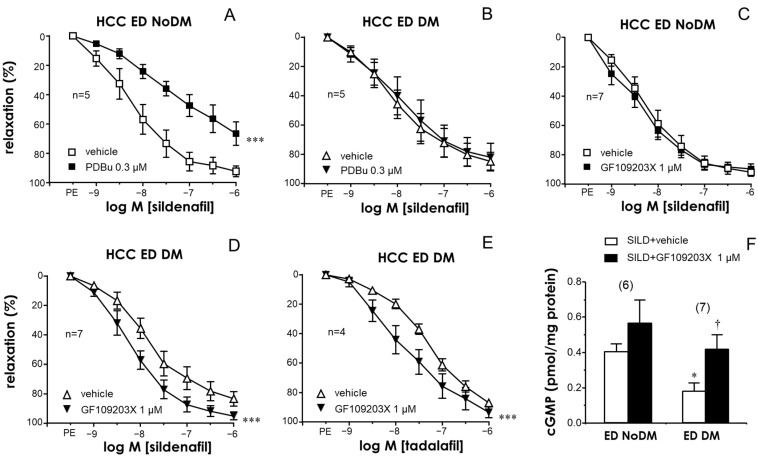
PKC inhibition enhanced the efficacy of PDE5 inhibition in relaxing the human corpus cavernosum and accumulating cGMP in diabetes. The effects of PKC activation with 12,13-phorbol dibutyrate (PDBu, 0.3 µM) (**A**,**B**) and of PKC inhibition with GF109203X (1 µM) (**C**,**D**) on relaxations induced by the PDE5 inhibitor sildenafil (1 nM to 1 µM) in the HCC of non-diabetic patients with ED (ED NoDM) (**A**,**C**) and diabetic patients with ED (ED DM) (**B**,**D**). (**E**) shows the effect of the PKC inhibitor on relaxations induced by the PDE5 inhibitor tadalafil (1 nM to 1 µM) in the HCC of ED DM patients. All tissues were precontracted with phenylephrine (PE, 1–10 µM). Data are expressed as the mean ± S.E.M of the percentage of relaxation. n indicates the number of patients from whom the tissues were collected. *** *p* < 0.001 according to a two-factor ANOVA. (**F**) shows the effects of GF109203X (1 µM) on the cGMP accumulation driven by sildenafil (SILD, 1 µM) in the HCC of ED NoDM and ED DM patients. Data are expressed as the mean ± S.E.M. of the pmoles of cGMP normalized by the protein content of the tissue. Numbers of patients are indicated in parentheses. * *p* < 0.05 vs. ED NoDM, † *p* < 0.05 vs. the respective data without GF109203X according to the Kruskal–Wallis test followed by Dunn’s post-hoc test.

A significant reduction in the relaxant efficacy of the PDE5 inhibitor sildenafil was observed in the CC and PRAs of diabetic patients with ED with respect to those of ED patients without diabetes (Supplementary Figure S5A,B). Similarly to the endothelial responses, the activation of PKC with PDBu (0.3 µM) reduced the relaxation capacity of the PDE5 inhibitor sildenafil in the CC of non-diabetic ED patients (Figure 4A) but not in cavernosal strips from diabetic ones (Figure 4B). Conversely, the inhibition of PKC with GF109203X (1 µM) produced a significant improvement of sildenafil-induced relaxations in the HCC of ED patients only when diabetes was present (Figure 4C,D). Potentiating effects by GF109203X were also confirmed when evaluating relaxations in response to another PDE5 inhibitor, tadalafil, in the CC of diabetic ED patients (Figure 4E). Furthermore, while treatment with GF109203X did not significantly modify cGMP accumulation driven by sildenafil in CC strips from non-diabetic ED patients, the reduction in the capacity of sildenafil to generate cGMP accumulation in the CC of diabetic ED patients was reversed by pretreating the strips with the PKC inhibitor (Figure 4F).

**Figure 5:**
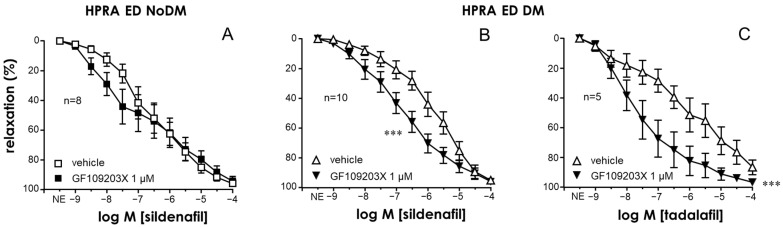
PKC inhibition enhanced the efficacy of PDE5 inhibition in causing vasodilation of human penile resistance arteries (HPRAs) in diabetes. Effects of PKC inhibition with GF109203X (1 µM) on vasodilations induced by the PDE5 inhibitor sildenafil (1 nM to 10 µM) in the HPRAs of non-diabetic patients with ED (ED NoDM) (**A**) and diabetic patients with ED (ED DM) (**B**). (**C**) shows the effect of the PKC inhibitor on relaxations induced by the PDE5 inhibitor tadalafil (1 nM to 100 µM) in the HCC of ED DM patients. All vascular preparations were precontracted with norepinephrine (NE, 1–10 µM). Data are expressed as the mean ± S.E.M of the percentage of relaxation. n indicates the number of patients from whom the tissues were collected. *** *p* < 0.001 according to a two-factor ANOVA.

PKC inhibition with GF109203X also failed to significantly enhance sildenafil-induced relaxations in the PRAs of non-diabetic ED patients (Figure 5A). In contrast, in the PRAs of diabetic ED patients, the treatment with the PKC inhibitor resulted in significant potentiation of the relaxant capacity of the PDE5 inhibitors sildenafil (Figure 5B) and tadalafil (Figure 5C). In fact, the treatment with GF109203X normalized sildenafil-induced relaxations in the CC and PRAs of diabetic ED patients, yielding relaxant responses that were not different from those obtained in strips from non-diabetic ED patients (Supplementary Figure S5C,D).

### 2.4. Specific Inhibition of the PKCβ2 Isoform Improved Endothelial Relaxation in the CC and PRAs of Diabetic ED Patients

**Figure 6:**
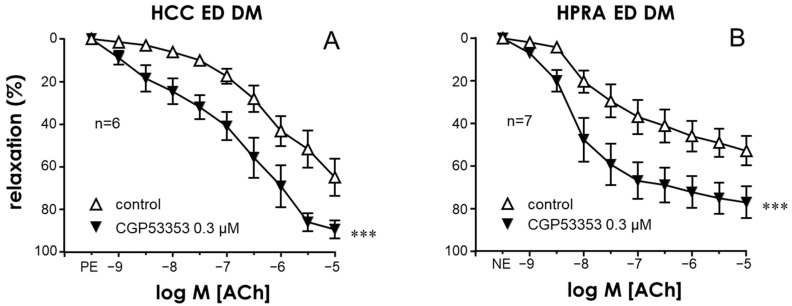
Specific inhibition of the PKCβ2 isoform improved endothelial relaxation of penile vascular tissues in diabetes. Effects of PKCβ2 inhibition with CGP53353 (0.3 µM) on endothelial relaxations in response to acetylcholine (ACh, 1 nM to 10 µM) in the human corpus cavernosum (HCC) (**A**) and penile resistance arteries (HPRAs) (**B**) of diabetic patients with ED (ED DM). HCC strips were precontracted with phenylephrine (PE, 1–10 µM), while HPRA segments were precontracted with norepinephrine (NE, 1–10 µM). Data are expressed as the mean ± S.E.M of the percentage of relaxation. n indicates the number of patients from whom the tissues were collected. *** *p* < 0.001 according to a two-factor ANOVA.

To identify the PKC isoform responsible for the endothelial impairment related to diabetes in the human corpus cavernosum, the effects of the specific inhibitor of the PKCβ2 isoform CGP53353 (0.3 µM) on endothelium-dependent relaxations in response to Ach were evaluated in CC strips and PRA segments from patients with diabetic ED. Treatment of penile vascular tissues with CGP53353 resulted in a significant potentiation of the endothelial relaxations in both the CC and PRAs of diabetic patients with ED (Figure 6A and Figure 6B, respectively), suggesting an involvement of this specific PKC isoform in diabetes-related endothelial impairment.

### 2.5. NADPH-Oxidase Was Involved in the Endothelial Impairment Caused by PKC in Penile Vascular Tissues

**Figure 7:**
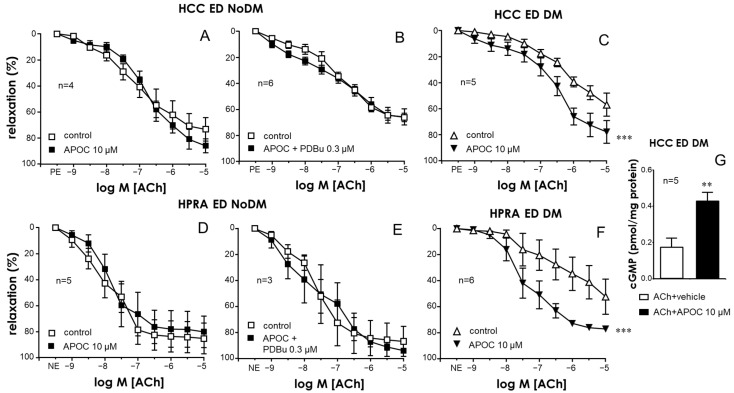
Inhibition of NADPH-oxidase improved endothelial relaxation and cGMP accumulation in the penile vascular tissues of diabetic patients and prevented the impairment of endothelial relaxations driven by PKC activation in non-diabetic ED. Effects of NADPH-oxidase inhibition with apocynin (APOC, 10 µM) on endothelial relaxations in response to acetylcholine (ACh, 1 nM to 10 µM) in the human corpus cavernosum (HCC) (**A**–**C**) and penile resistance arteries (HPRAs) (**D**–**F**) of non-diabetic patients with ED (ED NoDM) (**A**,**D**) and diabetic patients with ED (ED DM) (**C**,**F**). Panels B and E show the influence of the NADPH-oxidase inhibitor on the impairment driven by the PKC activator 12,13-phorbol dibutyrate (PDBu 0.3 µM) on endothelial relaxations induced by ACh in the HCC (**B**) and HPRAs (**E**) of ED NoDM patients. HCC strips were precontracted with phenylephrine (PE, 1–10 µM), while HPRA segments were precontracted with norepinephrine (NE, 1–10 µM). Data are expressed as the mean ± S.E.M of the percentage of relaxation. n indicates the number of patients from whom the tissues were collected. *** *p* < 0.001 according to a two-factor ANOVA. (**G**) shows the effects of APOC (10 µM) on the cGMP accumulation driven by ACh (10 µM) in the HCC of ED DM patients. Data are expressed as the mean ± S.E.M. of the pmoles of cGMP normalized by the protein content of the tissue. n indicates the number of patients. ** *p* < 0.01 according to the Mann–Whitney U-test.

The inhibition of NADPH-oxidase with apocynin (10 µM) did not modify ACh-induced relaxations in the CC or PRAs of non-diabetic ED patients (Figure 7A and Figure 7D, respectively). In contrast, apocynin treatment was able to significantly enhance endothelial relaxations in the CC and PRAs of diabetic ED patients, where an upregulated PKC was functionally presumed (Figure 7C and Figure 7F, respectively). Furthermore, the aforementioned impairment of ACh-induced relaxations caused by activating PKC with PDBu (0.3 µM) was prevented by co-treating with the NADPH-oxidase inhibitor in the CC and PRAs of non-diabetic ED patients (Figure 7B,E). The potentiation of endothelial relaxations was associated with a significant increase in ACh-induced cGMP due to apocynin in the CC of diabetic ED patients (Figure 7G).

## 3. Discussion

The present results suggest that PKC contributes to the functional impairment of penile vascular tissue in diabetic patients with ED. This is based on functional evidence showing that PKC inhibition specifically improved endothelium-dependent, neurogenic, and PDE5-inhibition-induced relaxations in human penile vascular tissues from diabetic patients by increasing cGMP accumulation. The PKCβ2 isoform seems to specifically contribute to diabetes-associated vascular alterations, as suggested by the improvements due to a specific inhibitor of this isoform. Moreover, NADPH-oxidase appears to be involved in the PKC-induced vascular effects on diabetic penile tissue, as indicated by the antagonizing effect of an NADPH-oxidase inhibitor, apocynin, on endothelial impairment driven by the activation of PKC in non-diabetic tissues and by its enhancing effect on such responses in penile vascular tissues in diabetic patients.

Erection is a neurovascular process that requires adequate neurogenic triggering and preserved vascular endothelium function to promote arterial vasodilation for blood inflow and cavernosal relaxation for the corporo-veno occlusive mechanism, which leads to blood accumulation and penile rigidity. Thus, inadequate function of neurovascular penile structures may result in ED [19]. In this sense, there is a large amount of evidence relating the presence of diabetes to vascular dysfunctions and neuropathies [20]. Indeed, microvascular and macrovascular complications are the leading cause of death in diabetic patients [21]. ED is highly prevalent among diabetic men [22], even in those with recent-onset diabetes [23]. ED is diagnosed 5–10 years earlier in diabetic than in non-diabetic men, suggesting an accelerated onset of ED in diabetes [24]. In fact, Mendelian randomization of genome-wide association studies suggested that type 2 diabetes has a direct causal effect on ED [25,26]. However, from a clinical viewpoint, the main challenge is that diabetic men represent a difficult-to-treat population of ED patients, since their therapeutic response to conventional treatment with PDE5 inhibitors is reduced [27,28]. This is probably related to a highly defective NO/cGMP pathway in penile vascular tissue in diabetic men with ED [5]. Thus, pharmacological strategies aiming to relieve the impairment of NO-/cGMP-mediated responses could have relevance in the management of diabetic ED.

Several mechanisms have been proposed to participate in vascular dysfunction related to hyperglycemia and diabetes. Among them, oxidative stress and PKC have been highlighted as outstanding contributors [10,11]. In this sense, chronic inhibition of PKCβ in diabetic mice resulted in an improvement of endothelium-dependent and neurogenic relaxations of the corpus cavernosum [12]. Furthermore, we previously reported an enhancement of endothelial relaxations of the human corpus cavernosum from diabetic ED patients by inhibiting PKC with GF109203X, suggesting that elevated PKC activity counteracted endothelial responses in diabetic ED [13]. The present results confirmed this idea by adding the evidence that the activation of PKC activity by phorbol 12,13-dibutyrate (PDBu) only impaired endothelial relaxations in the CC of ED patients without diabetes but not in that of diabetic ED patients, while the inhibition of PKC with GF109203X improved endothelial relaxation only in diabetic tissues. Then, the presence of an overactive PKC in the CC of diabetic ED patients would explain the lack of an effect due to the pharmacological activation of PKC and the improvement caused by PKC inhibition only in diabetic tissues, despite the fact that the CC of non-diabetic ED patients displayed endothelial impairment with respect to healthy tissues. Moreover, this functional evidence was supported by the significant increase in cGMP content in response to ACh that was only detected in the cavernosal tissue of diabetic ED patients, pointing to PKC overactivity specifically accounting for the impairment of the NO/cGMP pathway in diabetic ED. The results obtained in the CC were corroborated in the penile resistance arteries, another penile vascular structure that is fundamental in the hemodynamic process of erection. Although impairment of endothelial vasodilation was detected in the PRAs of non-diabetic ED patients, PKC inhibition was able to improve endothelial responses only in the arteries of diabetic ED patients, similarly to the observation in the CC. In addition, NO-independent vasodilation attributed to endothelial hyperpolarization was not modified by PKC inhibition in the PRAs of diabetic ED patients, indicating a specific involvement of the NO/cGMP pathway in the impact of PKC on penile vascular function in diabetes. This approach cannot be used in the CC because the inhibition of NO/cGMP in this tissue completely abrogates endothelial relaxation [8].

Neurogenic relaxation of the penile vasculature is essential for obtaining an erection. In this sense, neurogenic relaxation induced by electrical field stimulation in the CC under inhibiting conditions of cholinergic and adrenergic innervation is well known to be mediated by the release of NO due to parasympathetic innervation and is considered nitrergic [29,30]. Consistently with the impact of PKC overactivity on the NO/cGMP pathway related to diabetes, nitrergic relaxation was also enhanced by GF109203X in the CC of diabetic ED patients but not in that of non-diabetic ones. Thus, PKC inhibition improved both endothelial and neurogenic NO-mediated relaxations in the penile vascular tissues of diabetic ED patients, which are two fundamental events in the physiology of erection that are more profoundly impaired in ED patients when diabetes is present [5].

PDE5 limits NO-/cGMP-mediated responses by hydrolyzing cGMP. Thus, PDE5 inhibitors amplify NO/cGMP signaling by promoting cGMP accumulation in the tissue and enhancing the relaxation response [31,32]. This has led to the clinical use of PDE5 inhibitors for the treatment of ED, which remains the first-choice therapy for men with this condition [33,34]. However, as mentioned above, the clinical efficacy of these drugs is lower in diabetic patients [27,28], and this is related to the reduced capacity of PDE5 inhibitors to relax the CC and PRAs of these patients [35]. A role for PKC overactivity in this reduced relaxant activity of PDE5 inhibitors in diabetes was supported by the lack of influence of PKC activation on sildenafil-induced relaxations in the CC of diabetic ED patients, while only in these tissues, PKC inhibition resulted in enhanced relaxation in response to sildenafil. This specific effect of PKC modulation in diabetes was also observed in the PRAs, an effect that was reproduced when using a different PDE5 inhibitor, tadalafil, in both the CC and PRAs of diabetic patients with ED. Furthermore, this functional evidence was parallel to the ability of the PKC inhibitor to significantly increase the efficacy of sildenafil in inducing cGMP accumulation only in the CC of diabetic patients, which displayed reduced cGMP content in response to sildenafil. These results suggest that PKC overactivity could hinder the NO/cGMP pathway and contribute to the reduced efficacy of PDE5 inhibitors in causing penile vascular relaxation in diabetes, providing a rationale for PKC inhibition as a potential pharmacological strategy for potentiating the efficacy of PDE5 inhibitors in relieving ED in diabetes.

PKC comprises a family of isoforms that include classical PKC (α, β, and γ), which is activated by calcium and diacylglycerol (DAG) or phorbol esters, as well as other novel PKC and atypical PKC isoforms [11]. Although hyperglycemia-activated classical PKC and some other PKC isoforms have been postulated to contribute to diabetes-related complications [36], the PKCβ isoform has been consistently related to vascular complications in diabetes [37]. In fact, clinical trials have shown some beneficial effects on neurovascular complications in diabetes after administering the PKCβ inhibitor ruboxistaurin [38,39,40], including improvement in endothelial vasodilation [41]. GF109203X is a selective PKC inhibitor but has no clear specificity for PKC isoforms [42], although it has been shown to be a more potent inhibitor of the classical α and β isoforms [43]. In order to further delimit the PKC isoforms involved in diabetes-induced endothelial dysfunction of human penile vascular tissue, we evaluated the effects of a specific inhibitor of PKCβ2, CGP53353, which has been reported to inhibit inflammatory patterns induced by hyperglycemia in human endothelial cells [44]. The potentiating effects of CGP53353 on the endothelial relaxation of CC and PRAs from diabetic ED patients point to the involvement of PKCβ2 in the functional impact of diabetes on the human penile vasculature.

Oxidative stress plays a relevant role in the pathophysiology of diabetic ED [15]. In fact, the acute antioxidant approach has been shown to improve the endothelial and neurogenic relaxation of the CC and PRAs of diabetic patients with ED [45]. Oxidative stress and PKC activation are bidirectionally related, since ROSs are thought to cause PKC activation, while PKC activity is known to induce NADPH-oxidase-dependent ROS generation in vascular tissue in response to inflammatory stimuli and hyperglycemia [17,18,46]. Indeed, the PKCβ2 isoform has been found to be specifically involved in the PKC–NADPH-oxidase axis to promote oxidative stress [18,47]. In this sense, NADPH-oxidase hyperactivation has been proposed as responsible for oxidative stress related to diabetic ED [48]. The inhibition of NADPH-oxidase with apocynin [49,50,51] resembles the effects of PKC inhibition on endothelial relaxation in the CC and PRAs of ED patients, i.e., it only causes endothelial improvement in tissues of diabetic patients, by increasing cGMP content. This would be compatible with a pathophysiological mechanism consisting of PKC-dependent ROS generation through the activation of NADPH-oxidase that impairs the NO/cGMP pathway in penile vascular tissues in diabetes. This concept is further supported by the ability of apocynin to prevent the deleterious effect of PKC activation with PDBu on endothelial relaxation of both the CC and PRAs of non-diabetic ED patients.

The limitations of this study include the lack of biochemical determinations of PKC and NADPH-oxidase activity or ROS detection in human penile tissue. However, the results are strengthened by the extensive functional evidence obtained with contrasting inhibitors and activators of relevant enzymes using both cavernosal and penile arterial human tissues from a significant number of patients.

## 4. Materials and Methods

### 4.1. Human Tissues

Human corpus cavernosum (CC) biopsies were obtained from 76 patients with ED who gave informed consent at the time of insertion of a penile prosthesis. HCC specimens collected from 14 deceased organ donors (No ED) at the time of organ donation for transplantation whose relatives gave informed consent were also obtained. Patients with active infectious diseases or who were unable to provide informed consent were excluded. Tissues were maintained from 4 °C to 6 °C in M-400 solution (composition per 100 mL: mannitol, 4.19 g; KH_2_PO_4_, 0.205 g; K_2_HPO_4_·3H_2_O, 0.97 g; KCl, 0.112 g; NaHCO_3_, 0.084 g; pH 7.4) until they were used, which occurred in a range of 16 to 24 h after extraction [5,6,35]. Protocols and consent forms were approved by the Ethics Committees of Hospital Universitario Ramón y Cajal in Madrid, Spain (Ethics Approval Procedure 16/045) and Hospital Santo Antonio in Porto, Portugal (210-15 (174-DEFI/156-CES). Samples from ED patients were divided into two groups depending on the presence (diabetic ED, n = 36) or the absence (non-diabetic ED, n = 40) of diabetes according to the information from clinical records.

#### 4.1.1. Functional Evaluation of CC

Cavernosal tissue specimens were divided into strips (approximately 3 mm × 3 mm × 7 mm) and immersed in 8 mL organ chambers containing Krebs–Henseleit solution (which was comprised of the following composition (mM): NaCl 119, KCl 4.6, CaCl2 1.5, MgCl_2_ 1.2, NaHCO_3_ 24.9, glucose 11, KH_2_PO_4_ 1.2, EDTA 0.027) at 37 °C while being continuously bubbled with a 95% O_2_/5% CO_2_ mixture to maintain a pH of 7.4 for the recording of isometric tension, as previously described [5,6,35]. Each tissue strip was incrementally stretched to the optimal isometric tension as determined by the maximal contractile response to 1 µM phenylephrine (PE). The preparations were then exposed to a high K^+^ concentration (125 mM), and the contractile response was measured. Endothelium-dependent relaxation responses were evaluated by using cumulative additions of ACh (1 nM to 10 µM) on cavernosal strips precontracted with PE (1–10 µM, approximately 80% of K^+^-induced contraction). The responses were evaluated before (control) or after treating the tissues for 60 min with the PKC activator 12,13-phorbol dibutyrate (PDBu, 0.3 µM), the PKC inhibitor GF109203X (1 µM), a specific inhibitor of PKCβ2 isoform (CGP53353, 0.3 µM), the NADPH-oxidase (NOX) inhibitor apocynin (10 µM), or a vehicle (dimethyl sulfoxide, DMSO, 0.1%). Relaxation responses to type 5 phosphodiesterase inhibition were determined by cumulatively adding sildenafil (1 nM to 1 µM) or tadalafil (1 nM to 1 µM) on PE-contracted strips of the HCC. The responses were evaluated after 60 min of exposure to the vehicle, PDBu, or GF109203X.

Electrical field stimulation was applied to CC strips contracted with PE by means of two platinum electrodes placed at both sides of the tissue and connected to a current stimulator (Cibertec, Madrid, Spain). The parameters of electrical field stimulation were 75 mA and 0.5 ms for 20 s. Neurogenic nitrergic relaxations to electrical field stimulation (0.5 to 16 Hz) were obtained in CC strips treated with guanethidine (30 µM) and atropine (0.1 µM) (to inhibit NE release and muscarinic receptor activation, respectively, in order to block adrenergic and cholinergic neurotransmission to allow the evaluation of nitrergic responses) [5,45]. Electrical-field-stimulation-induced relaxations were performed under control conditions and after exposure to the vehicle, PDBu, or GF109203X. All drugs were obtained from Sigma-Aldrich (Saint Louis, MO, USA), except for CGP53353, which was obtained from Tocris Bioscience (Bristol, UK).

#### 4.1.2. Functional Evaluation of Human Penile Resistance Arteries (PRAs)

Penile small helicine arteries (lumen diameter: 150 µm to 500 µm), which are the terminal branches of deep penile arteries, were dissected from the CC specimens by carefully removing the adhering cavernosal tissue. Arterial ring segments (1.7–2.0 mm long) were subsequently mounted on microvascular wire myographs (Danish MyoTechnology; Aarhus, Denmark) for isometric tension recordings [5,6,35]. The vessels were allowed to equilibrate for 30 min in Krebs–Henseleit solution at 37 °C and were continuously bubbled with a 95% O_2_/5% CO_2_ mixture to maintain a pH of 7.4. The passive tension and internal circumference of the vascular segments when relaxed in situ under a transmural pressure of 100 mm Hg (L_100_) were determined. The arteries were then set to an internal circumference equivalent to 90% of L_100_, at which the force development was close to maximal. The preparations were then exposed to high K^+^, and the contractile response was measured. PRA segments that failed to produce a tension equivalent to a pressure of 100 mmHg were rejected. Endothelium-dependent relaxation responses were evaluated by using cumulative additions of ACh (1 nM to 10 µM) on penile arterial segments that were precontracted with norepinephrine (NE, 1–10 µM, approximately 80% of K^+^-induced contraction). The responses were evaluated before (control) or after treating the arteries for 60 min with PDBu, GF109203X, CGP53353, apocynin, or the vehicle. Relaxation responses to type 5 phosphodiesterase inhibition were determined by cumulatively adding sildenafil (1 nM to 100 µM) or tadalafil (1 nM to 100 µM) on NE-contracted PRA. The responses were evaluated after 60 min of exposure to the vehicle, PDBu, or GF109203X.

#### 4.1.3. Cyclic GMP Determinations

CC strips immersed in organ baths were treated for 60 min with GF109203X, apocynin, or the vehicle and exposed to ACh (10 µM) or sildenafil (1 µM) for 5 min. Tissues were then snap-frozen in liquid nitrogen and stored at −80 °C until the determination of cyclic GMP (cGMP). Tissues were extracted through homogenization in 6% trichloroacetic acid, followed by ether (H_2_O-saturated) extraction and lyophilization. The concentration of cGMP was determined with an enzyme-linked immunosorbent assay by using a kit from the Cayman Chemical Company (Ann Arbor, MI, USA) [35].

### 4.2. Data Analysis

The data are expressed as the mean ± standard error. For comparison of complete concentration–response curves, a two-factor analysis of variance (ANOVA) test was applied. This statistical test compared the concentration–response curves in their entirety, including all concentrations in the analysis. When multiple concentration–response curves were compared, Bonferroni’s correction was applied. For other multiple comparisons, the Kruskal–Wallis test was used, followed by Dunn’s post-hoc test. Discrete variables were compared by using Fisher’s exact test, while all other data were compared by using a t-test or Mann–Whitney U-test, when appropriate, with GraphPad Prism software 6.0 (San Diego, CA, USA). A probability of <0.05 was considered significant.

## 5. Conclusions

**Figure 8:**
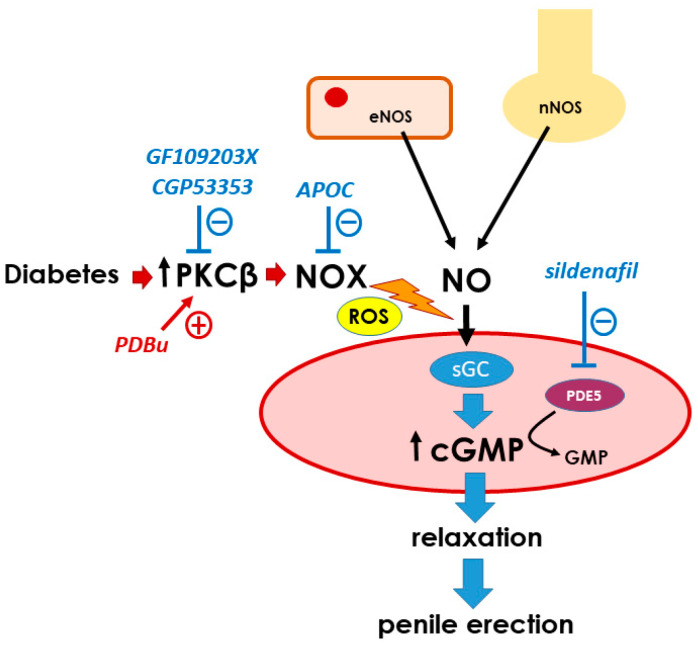
Schematic representation of the potential effects of PKC inhibition on diabetic erectile dysfunction. Nitric oxide released upon sexual stimulation from the endothelium (through endothelial nitric oxide synthase, eNOS) and nitrergic nerves (through neuronal NOS, nNOS) stimulates soluble guanylyl cyclase (sGC) in penile smooth muscle, which increases cyclic guanosine monophosphate (↑cGMP), leading to relaxation of the smooth muscle and penile erection. Type 5 phosphodiesterase (PDE5) is the main enzyme regulating the hydrolysis of cGMP to GMP in penile smooth muscle. PDE5 inhibitors (−) such as sildenafil amplify NO-mediated signaling by slowing cGMP degradation and represent the first-line therapy for erectile dysfunction (ED). However, these drugs require some level of NO availability for their therapeutic effect, which might be compromised in diabetic ED, increasing the risk of treatment failure. The impact of diabetes seems to be related to increased protein kinase C-β (↑PKC-β) activity, since the inhibition of PKC with GF109203X, and, specifically, PKC-β with CGP53353 results in enhanced NO-mediated functional responses, including PDE5-inhibitor-induced relaxation, only in penile tissues from diabetic patients. PKC would increase the generation of reactive oxygen species (ROSs) by NADPH-oxidase (NOX), reducing NO bioavailability and, thus, compromising penile erection. This is supported by the ability of the NOX inhibitor apocynin (APOC) to improve NO-mediated responses in penile tissues of diabetic men and to block the deleterious effect on this response driven by the pharmacological stimulation (+) of PKC in non-diabetic tissues with 12,13-phorbol dibutyrate (PDBu).

This study evidenced that PKC inhibition improved the endothelial and neurogenic responses of penile vascular tissues of diabetic patients with ED, relieving the impairment of the NO/cGMP pathway in these tissues and suggesting PKC overactivity as a specific pathophysiological mechanism of human diabetic ED. This mechanism probably involves ROS generation through PKC-dependent hyperactivation of NADPH-oxidase, although further studies are warranted to obtain molecular evidence. By recovering the NO/cGMP pathway, PKC inhibition results in augmented efficacy of PDE5 inhibitors in relaxing the penile vascular tissues of diabetic patients, and this approach is proposed as a potential pharmacological target to increase the number of diabetic patients displaying therapeutic responses to ED treatments (Figure 8).

## Figures and Tables

**Table 1:** Characteristics of patients with erectile dysfunction.

	Non-Diabetic	Diabetic	*p* Value
n	40	36	
Age (years)	59.1 ± 1.6	57.5 ± 1.7	0.4666
Hypertension (%)	18 (45.0)	17 (47.2)	1.0000
Dyslipidemia (%)	7 (17.5)	10 (27.8)	0.4090
Obesity (%)	4 (10.0)	1 (2.8)	0.3616
Smoking habit (%)	14 (35.0)	6 (16.7)	0.1164
Cardiovascular disease (%)	1 (2.5)	5 (13.9)	0.0954
Respiratory disease (%)	2 (5.0)	2 (5.6)	1.0000
Pelvic surgery (%)	11 (27.5)	7 (19.4)	0.4338
Peyronie’s disease (%)	3 (7.5)	2 (5.6)	1.0000

n indicates the number of subjects. Smoking habit refers to a present or former habit. Age is expressed as the mean ± S.E.M. and was compared by using the Mann–Whitney U-test. Other variables were compared between the two groups by using Fisher’s exact test. All subjects underwent penile prosthesis insertion due to erectile dysfunction either without or with diabetes.

## Data Availability

The data presented in this study are available on request from the corresponding author. The data are not publicly available due to data protection policy.

## Supplementary Materials

The following supporting information can be downloaded at: https://www.mdpi.com/article/10.3390/ijms25063111/s1.
