# Supplementary figures and images for: PKC Inhibition Improves Human Penile Vascular Function and the NO/cGMP Pathway in Diabetic Erectile Dysfunction: The Role of NADPH Oxidase
Source: Int J Mol Sci. 2024 Mar 7;25(6):3111. doi: 10.3390/ijms25063111 (PMC10970662; doi:10.3390/ijms25063111)

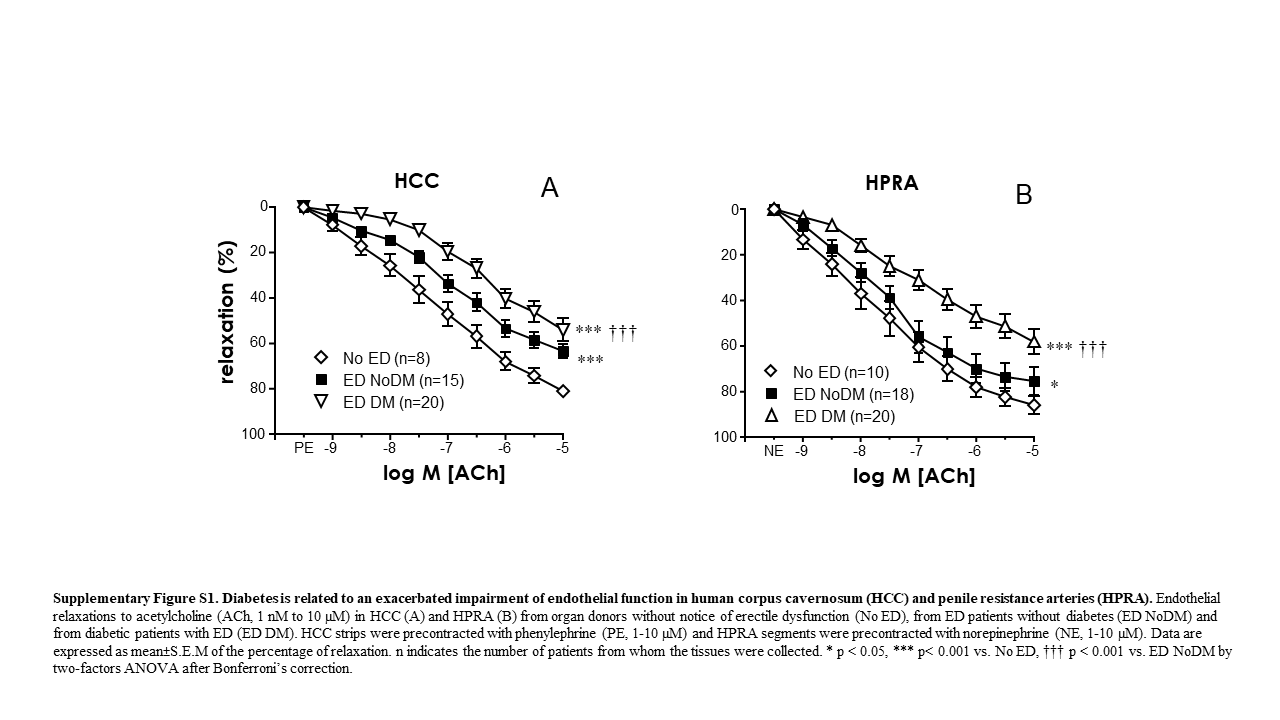

Supplement: Supplementary file 1 [file ijms-25-03111-s001.zip › Supplementary Figure S1.TIF]

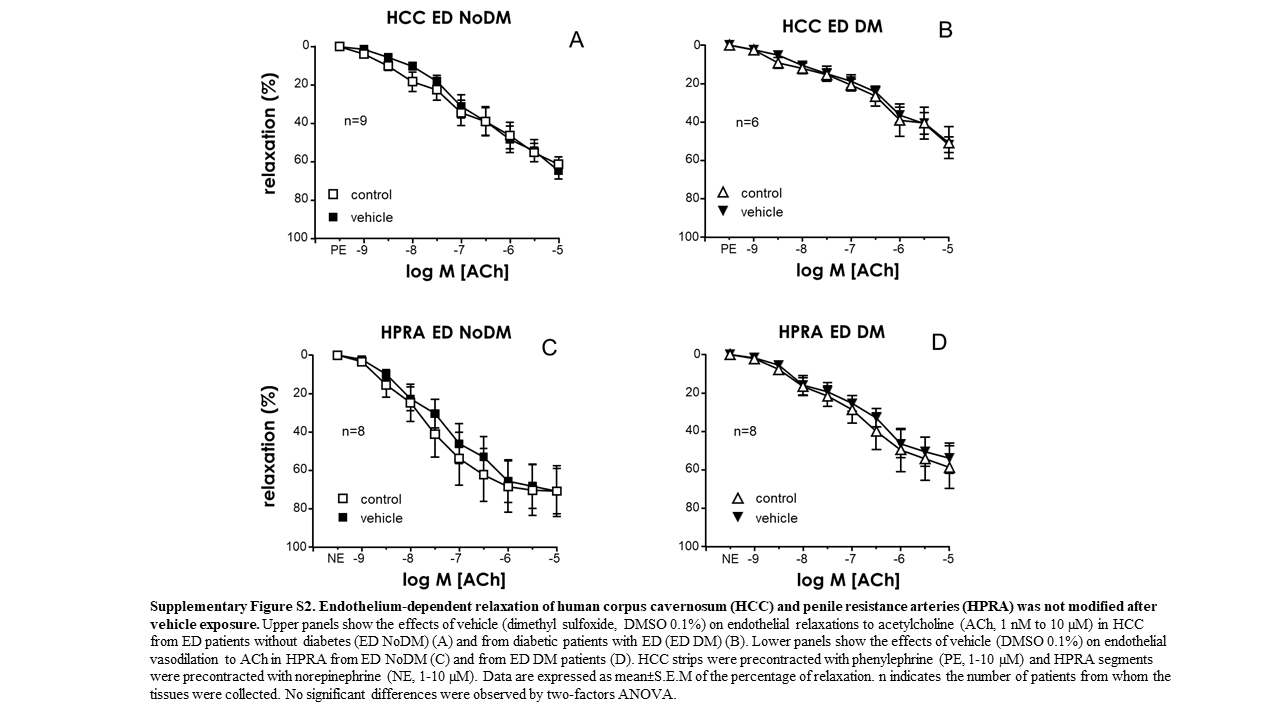

Supplement: Supplementary file 1 [file ijms-25-03111-s001.zip › Supplementary Figure S2.TIF]

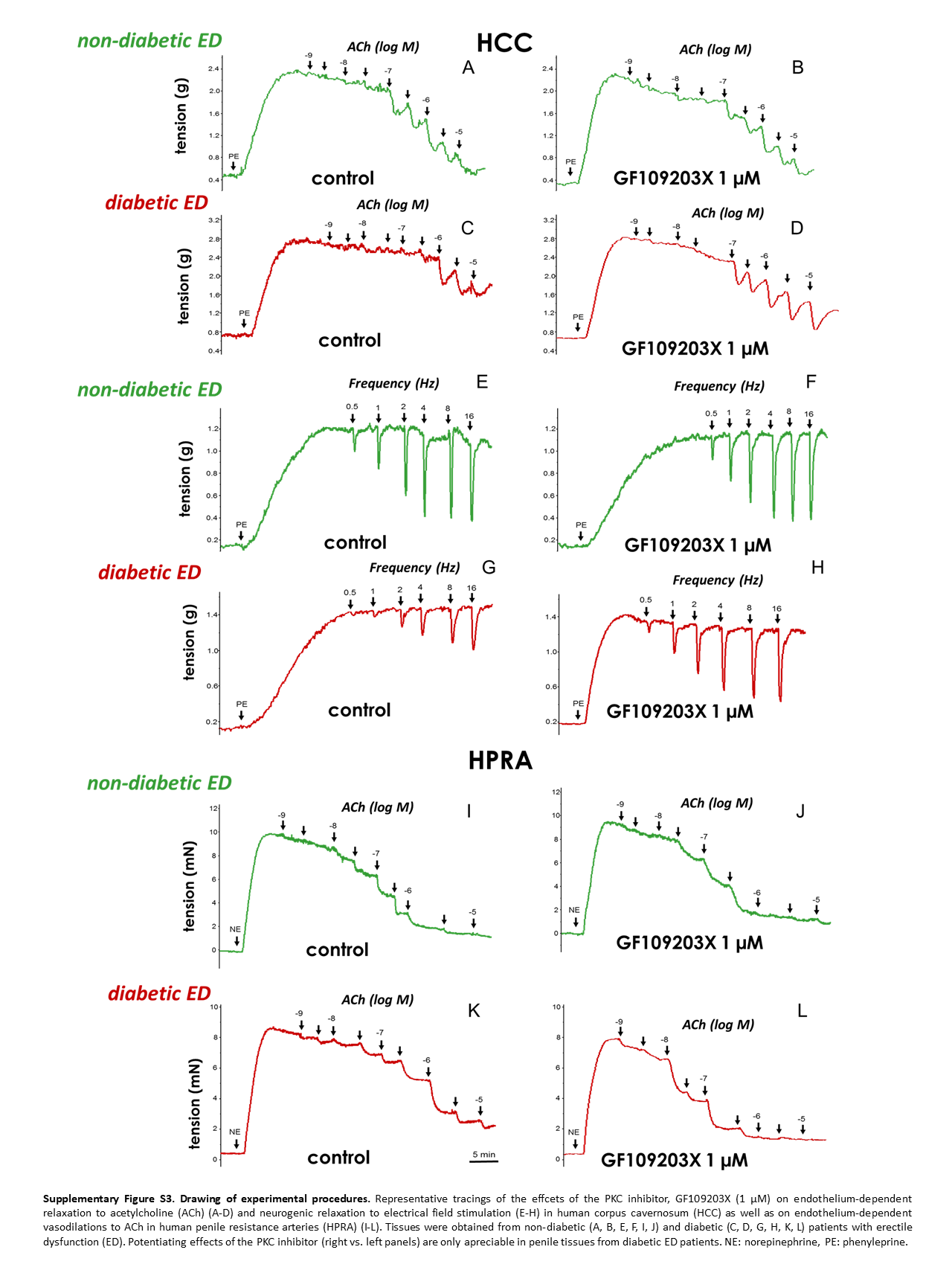

Supplement: Supplementary file 1 [file ijms-25-03111-s001.zip › Supplementary Figure S3.tif]

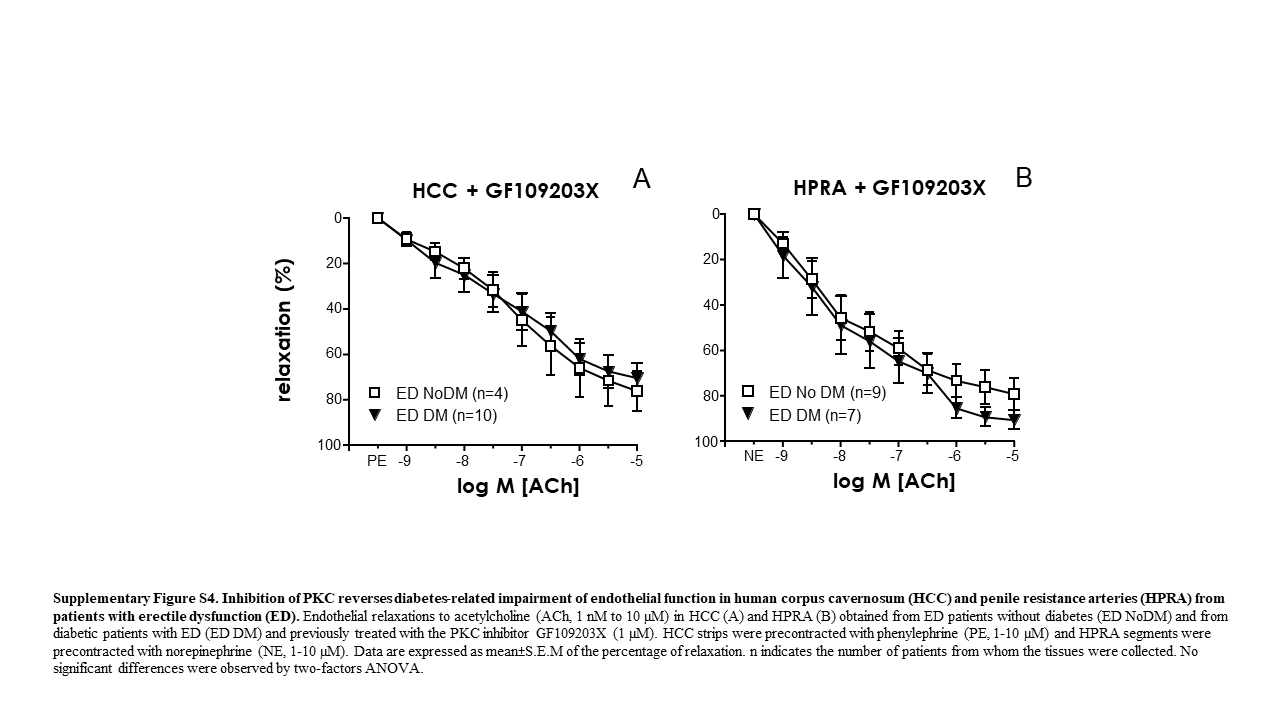

Supplement: Supplementary file 1 [file ijms-25-03111-s001.zip › Supplementary Figure S4.TIF]

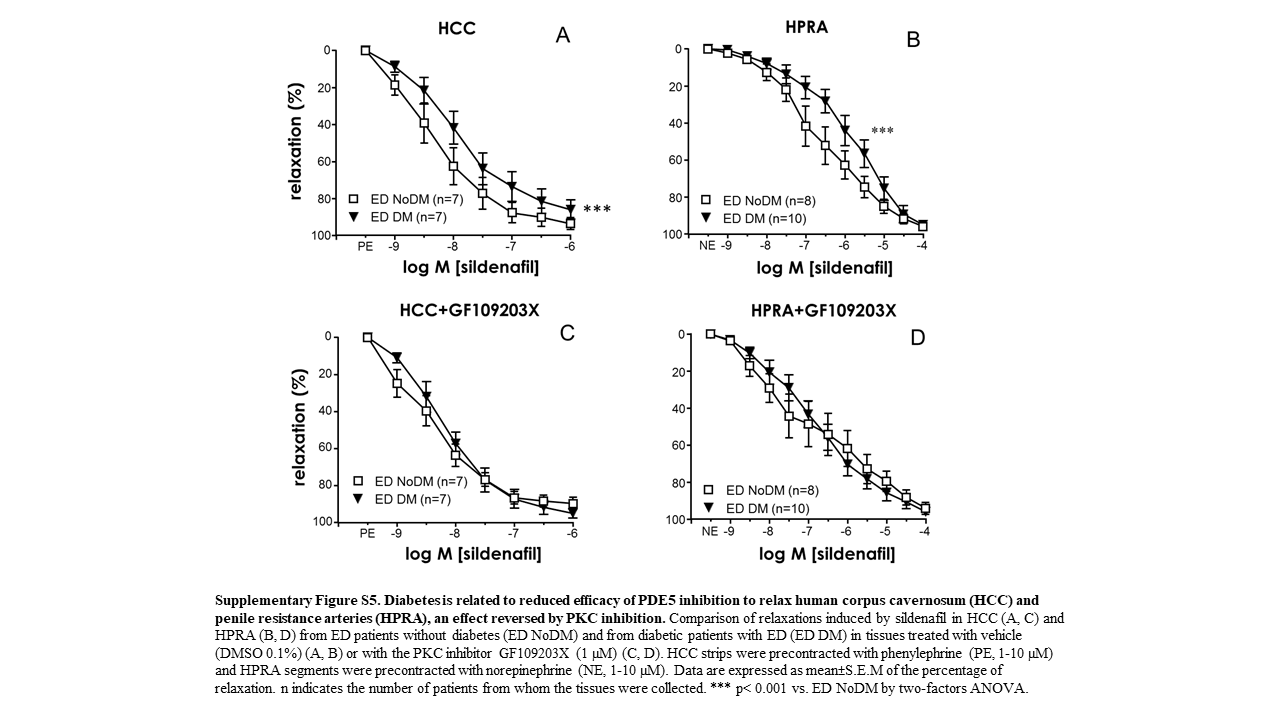

Supplement: Supplementary file 1 [file ijms-25-03111-s001.zip › Supplementary Figure S5.TIF]
